# Status of food safety knowledge, attitude, and practices of caregivers of children in northern Uganda

**DOI:** 10.1002/fsn3.3504

**Published:** 2023-06-28

**Authors:** Eunice Achiro, Lawrence Okidi, Richard Echodu, Simon Peter Alarakol, Prossy Nassanga, Duncan Ongeng

**Affiliations:** ^1^ Department of Food Science and Postharvest Technology, Faculty of Agriculture and Environment Gulu University Gulu Uganda; ^2^ Department of Biology, Faculty of Science Gulu University Gulu Uganda; ^3^ Department of Medical Biochemistry, Faculty of Medicine Gulu University Gulu Uganda

**Keywords:** behavior, caregivers, complementary foods, food hygiene, food safety, homemade

## Abstract

The safety of homemade weaning foods in low‐ and middle‐income countries is of great concern as rural households have limited access to standardized commercial weaning foods. In the Acholi subregion of Uganda, complementary foods are locally produced. However, there is limited information on the Food safety knowledge (FSK), food safety attitude (FSA), and food hygiene practices (FHP) of the caregivers. This study examined food safety knowledge, attitude, and practices of the caregivers of children 6–23 months of age in Amuru and Nwoya districts, Northern Uganda, between March 2019 and June 2019. A cross‐sectional study was conducted involving 180 caregivers. Data were collected using semi‐structured questionnaires and focus group discussions and analyzed using descriptive statistics, multivariate binary logistic regression, and thematic content analysis. Caregivers had sufficient FSK (74.1%) and positive FSA (68.1%). However, only 17.6% of them adhered to FHP. Frequency of food safety training (*p* = .041) and households with children who suffered from foodborne illness (*p* = .001) significantly predicted FSK. Conversely, both FSK and FSA were significantly predicted by gender roles in decision‐making on household income (*p* = .006) and households with older children (*p* = .041). A significant positive correlation was observed between FSK and FSA (*r* = .406, *p* = .000). However, major barriers to adherence to FHP were inadequate sanitation facilities and caregiver's workload. The overall nontranslation of sufficient FSK and positive FSA into proper FHP calls for future intervention to harness the sociodemographic factors that influence FSK and FSA and address the barriers to FHP among caregivers.

## Graphical abstract

The safety of homemade weaning foods in developing countries is of great concern as rural households have limited access to standardized commercial weaning foods. In the Acholi subregion of Uganda, complementary foods are locally produced, and caregivers of children had sufficient FSK (74.1%) and positive FSA (68.1%) but only 17.6% of them adhered to FHP. Major barriers to adherence to FHP were inadequate sanitation facilities and caregiver's workload.

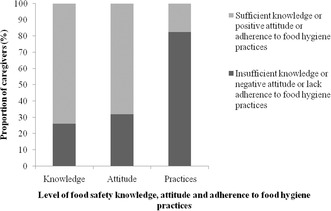

## 1 INTRODUCTION

Food safety is a universal precondition necessary for the achievement of good complementary feeding outcomes among children 6–23 months of age (Issaka et al., 2014). This is particularly because complementary foods are introduced at 6 months, a point in time at which breast milk solely cannot adequately support a child's nutrient needs for optimal growth despite the children having an underdeveloped immune system (Agostoni et al., 2009; Du Plessis et al., 2013). In many low‐ and middle‐income countries, contamination of complementary foods is one of the major factors that compromise the nutritional outcome of complementary feeding largely due to the occurrence of diarrheagenic foodborne diseases caused by nontyphoidal *Salmonella* and *Escherichia coli* among others (Scallan et al., 2015). As such, complementary feeding of children with safe food becomes a prerequisite for reducing diarrheagenic foodborne diseases and consequently attaining optimal growth among children (Sihag et al., 2015). Evidence in the literature shows that local production of safe complementary foods and their utilization necessitates that caregivers practice hygienic food behavior (Kunadu et al., 2016). This makes efforts to address food safety challenges, especially among caregivers of children 6–23 months from a behavioral viewpoint extremely important in realizing good nutrition outcomes and attainment of sustainable development goal 3; good health and well‐being (Nabyonga‐Orem, 2017).

Universally, foodborne illnesses (FBI) greatly threaten public health and socioeconomic progression due to the associated morbidity and mortality (Devleesschauwer et al., 2018). FBI is commonly caused by microbiological, chemical, and physical hazards resulting in vast health challenges. Among the foodborne hazards, microbiological hazards have been regarded as the major threat in recent years (Gizaw, 2019) and are caused by pathogenic viruses, bacteria, molds, yeast, protozoa, and their associated toxins (Lyashchuk et al., 2021; Vandeweyer et al., 2021). The presence of these microbiological agents in food beyond acceptable limits renders the food unsafe for human consumption (Bersisa et al., 2019; Negash, 2018; Saipullizan et al., 2018). Recent studies show that the biggest burden of FBI is attributable to pathogenic bacteria such as *E. coli*, *campylobacter*, norovirus, and nontyphoidal *Salmonella enterica* that cause diarrhea (Aworh, 2021; Chau et al., 2019; Lund, 2015). Globally, 40% of the burden of all FBI is on children under 5 years possibly due to their underdeveloped immune systems (Havelaar et al., 2015; Kloc et al., 2020). Although data on foodborne diseases are often scanty in the case of low‐ and middle‐income countries, data are often available in the form of foodborne disease‐associated manifestations such as diarrhea prevalence. For instance, a recent study in Uganda reported that 41% of cases of hospitalized children under 5 years had diarrhea resulting from *Salmonella* infection (Appiah et al., 2021). Particularly in northern Uganda, 4.9% of children under 5 years had cases of diarrhea (Uganda Bureau of Statistics (UBOS), 2019). The prevalence of diarrheal disease among children suggests that there is the existence of nonadherence to good food hygiene practices among caregivers. Indeed, studies on complementary feeding have shown that diarrheal disease incidence among children becomes more pronounced from the age of 6 months when complementary foods are introduced (Ogbo et al., 2018; Rao et al., 2011).

Owing to their low purchasing power of standardized hygienically produced complementary foods, households in rural areas of several low‐ and middle‐income countries largely rely on locally produced (homemade) complementary foods (Pelto et al., 2013). Even though locally produced complementary foods are widely acceptable, used, and nutritious (Alowo et al., 2018; Nassanga et al., 2018; Theurich et al., 2019), their microbiological safety is of major public health concern. However, it is important to note that the safety of such locally produced complementary food is largely dependent on the food‐handling practices of caregivers involved in their preparation and utilization (Wodnik et al., 2018). Thus, sanitary practices during the preparation of such products have to be strictly followed to minimize the occurrence of FBI among children fed on locally made complementary foods (Mshida et al., 2020; Teshome et al., 2022). Additionally, because most rural communities including those in northern Uganda produce complementary foods that are largely plant based comprising mainly cereals such as maize (*Zea mays*), millet (*Eleusine coracana*), and legumes such as soybeans (*Glycine max*) among others (Alowo et al., 2018; Lukwago et al., 2019), aflatoxin contamination may be another threat. A typical example is the malted millet–sesame–soy composite (MMSSC) that was recently formulated and is being promoted among caregivers of children aged 6–23 months in Acholi, a subregion of northern Uganda (Alowo et al., 2018). The aforementioned locally produced complementary foods are potentially prone to microbial contamination if FHP is not stringently adhered to. Therefore, to ensure that children 6–23 months are fed microbiologically safe, locally produced complementary foods, it is vital that caregivers have sufficient food safety knowledge and a positive attitude associated with food‐handling practices from the production to the consumption stage.

However, despite the reported high occurrence of foodborne diseases among children under 5 years (WHO, 2015), the caregivers' knowledge, attitude, and practices regarding food safety of locally produced complementary foods in the context of the rural environment have largely remained underinvestigated. Studies on the significance of knowledge, attitude, and practices on the outcome of complementary feeding have largely been focused on aspects associated with nutrient quality, quantity, and frequency (Aguayo et al., 2018; Pratik et al., 2017) and the elements of primary healthcare (Assefa et al., 2021; Chidziwisano et al., 2019; Gebresilasie et al., 2018; Hassan Khatib & Joho, 2022; Yimenu et al., 2022). This leaves a lacuna in the knowledge of whether caregivers who produce local complementary foods have sufficient food safety knowledge, positive attitude, and follow good FHP; which are essential in reducing the vulnerability of children to foodborne diseases (Vasco & Alvito, 2011). Thus, caregivers' knowledge, attitude, and practices toward the safety of complementary food formulae produced in rural domestic environments need to be adequately investigated. Hence, the objective of this study was to determine the status of food safety knowledge, attitude, and food hygiene practices of the caregivers of children aged 6–23 months utilizing MMSSC as complementary food and their predictors as well as barriers to FHP.

## 2 MATERIALS AND METHODS

### 2.1 Study design

This study adopted a cross‐sectional design involving a survey using in‐depth household interviews and focus group discussions (FGDs). The study was conducted in the Acholi subregion in Amuru (02°50′N 33°05′E) and Nwoya (02°38′N 32°00′E) districts, Northern Uganda, between March and June 2019. The two districts were selected to follow up on a study conducted by Alowo et al. (2018). The study population consisted of caregivers (mother/father of the child or legal guardian who takes care of the child in the absence of the mother/father) of children (6–23 months).

### 2.2 Sample size and sampling framework

A total of 180 participants who were used in the previous study conducted by Alowo et al. (2018) were recruited in this study. They included all caregivers who were trained on local production of MMSSC from two subcounties per district in the Nwoya and Amuru districts of northern Uganda. From the two purposively selected subcounties per district, one parish was purposively selected. In each parish, one village was then purposively selected. The inclusion criteria were a caregiver who received training on local production of MMSSC and was willing to participate in the study. The training of the caregivers lasted for 82 days and aimed at equipping them with good hygiene and good manufacturing practices to produce safe complimentary food composite in a domestic environment. The training content included the use of safe water and raw materials for making the composite, hand washing of both the caregiver and the child, keeping the utensils and environment clean, thorough cooking, and keeping cooked food covered in a safe place. The training was carried out in the local dialect (Acholi).

### 2.3 Data collection

A modified pretested questionnaire with provision for sociodemographic characteristics of the household adopted from FAO (2014) and Marías and Glasauer (2014) was used to assess the knowledge, attitude, and practices (KAP) of the caregivers. To attenuate the general answer bias according to Dunsch et al. (2018), 10 food safety knowledge questions were both positively and negatively framed. Also, six both positively and negatively framed questions were used for measuring attitude on a 3‐point Likert scale (0 = disagree, 1 = neither agree nor disagree, and 2 = agree) (Anand & Puri, 2013). Caregivers' self‐reported practices on the other hand were assessed by asking caregivers to articulately express how they handle food, especially food for complementary feeding from postharvest to consumption. To supplement the information obtained from household interviews, the community's perspective on the safety of MMSSC and food safety, in general, was obtained using the pretested FGDs guide based on health belief model constructs (Pourtaheri et al., 2018; Rosenstock, 1974; Rosenstock et al., 1988). Before household interviews and FGDs, modified semi‐structured questionnaires and FGD guide adopted from WHO (2010) and Marías and Glasauer (2014) were pretested among 30 caregivers of children aged 6–23 months attending postnatal care at Laroo health center III in Gulu district to ensure accuracy, clarity, and consistency in interpretation of questions. The responses were analyzed to check for validity and ambiguity. Results of the first pretest showed that respondents found difficulties interpreting the questions such as, “What is the source of birth?” This question was revised to “What is the place of birth?” Additionally, participants did not respond to the question, “What is the estimated household income allocated to food?” This question was later split into two questions to read “What is the estimated household income in a month?” and “What is the estimated amount spent on food?” The checked and validated pretested tools were then re‐pretested among 30 caregivers of children aged 6–23 months in Unyama Village, Gulu District (a similar distant community), within 1 week. From the second pretest, respondents did not have much difficulty and the responses were in line with the study objective. However, results from the second pretest showed that most (97%) respondents failed to estimate the money spent on food in a month. Therefore, this question was excluded from the data collection instrument.

Trained research assistants with experience in nutrition surveys and fluent in English and the local language (Acholi) administered the study questionnaires and conducted FGDs in the local dialect (Acholi). The study instruments were written in English and translated into the local dialect (Acholi). To guarantee confidentiality and prevent any interference, other members of the household and members of the neighboring household were not allowed to participate or be present during the interview and FGDs. And to ensure the originality of information, FGD consisted of 6 to 10 participants (caregivers of children aged 6–23 months) who had not participated in the household interview. As affirmed by Guest et al. (2006) and Mclafferty (2004), a total of eight FGDs were conducted which is above the minimum number of six FGDs required to yield saturation of information.

### 2.4 Data analysis

Statistical analyses were performed using the Statistical Package for Social Sciences (SPSS) version 21. For the case of knowledge questions, correct answers were scored 1, wrong answers, and in scenarios where the respondent did not know, a score of 0 was awarded. An attitude score of 0, 1, and 2 were given for disagreeing, neither agree nor disagree, and agree, respectively. Conversely, the reverse scores were given to negatively framed questions. This implied that 0, 1, and 2 were given for agreeing, neither agree nor disagree, and disagree, respectively (Anand & Puri, 2013). Knowledge and attitude for each caregiver were calculated by summing up scores on each question and the overall score ranked as sufficient or insufficient knowledge and positive or negative attitude, respectively. The cutoff for both sufficient knowledge and a positive attitude was 70% based on previously published studies (Tamang et al., 2020). Descriptive statistics (frequency, percentages, mean, and standard deviation) where applicable were used to summarize socioeconomic demographic characteristics and knowledge, attitude, and practices (KAP) data. To establish the socioeconomic and demographic predictors of sufficient knowledge and positive attitude, Pearson correlation analysis was run before regression analysis to eliminate highly correlated variables (*r* > .7) (Dormann et al., 2013). Multivariate binary logistic regression was then performed using Equation (1):

(1) $$Y_{i}=\alpha +\beta_{1}X_{1}+\beta_{2}X_{2}+\beta_{3}X_{3}\ldots \ldots \ldots .\beta_{17}X_{17}+\mu$$

where *Y*_*i*_ is the binary dependent variable representing the status of knowledge and attitude; *α* is the regression constant; *β* is the regression coefficient; *X*_1_ to *X*_17_ were the independent variables; and *μ* is the error term.

The independent variables selected to run the multivariate binary logistic regression are shown in Table 1. Binary multivariate logistic regression analysis was then performed to establish the sociodemographic predictors of sufficient knowledge and positive attitude. Significant statistical differences were considered at a 95% confidence level (*p* < .05). FGD data were transcribed, coded, and organized into common themes based on the health belief model constructs (Pourtaheri et al., 2018) and then analyzed using qualitative content analysis (Cho & Lee, 2014).

**TABLE 1:** Independent variables selected to run the multivariate binary logistic regression.

S/N	Social‐demographic variables	Responses
1	District/location	Nwoya = 1, Amuru = 2
2	Age of the caregiver	Years
3	Gender of the caregiver	1 = male, 0 = female
4	Education level of household head	1 = formal, 0 = non‐formal
5	Occupation of household head	1 = farmer, 0 = others
6	Marital status	1 = married, 0 = others
7	The main source of income	1 = sale of agricultural produce, 0 = others
8	Frequency of food safety training	1 = two or more times, 0 = once
9	Food safety training	1 = yes, 0 = no
10	Household size	Number of people
11	Households with children	1 = 1–17 years, 0 = children aged ≤5
12	Gender of the child	1 = male, 0 = female
13	Whether a child has ever suffered from symptoms of foodborne illness	1 = yes, 0 = no
14	Where the child was treated	1 = medical unit, 0 = others
15	Decision on family income	1 = male, 0 = others
16	Decision on food to cook	1 = male, 0 = male
17	Responsibility for provision for the household	1 = male, 0 = female

## 3 RESULTS

### 3.1 Sociodemographic characteristics of the caregivers

Table 2 shows that the female caregivers were three times more than the male caregivers. The majority (91.3%) of the caregivers were within the age bracket of 18–45 years with an average age of 31.5 years. The most predominant age group of the caregivers was between 18 and 25 years and three‐quarters of the caregivers were married. Three‐quarters of both the household heads and caregivers were subsistence farmers. Others were formally employed or casual laborers, but more caregivers (12%) were involved in casual work than household heads (9.5%). In terms of the level of education attained by the caregivers, a greater percentage attained a primary level of education (57%), followed by secondary (31.1%), no formal education (11.2%), and lastly tertiary education (1.7%). On average, the household size in this community was seven people. Families with household sizes less than seven were more than those with household sizes greater than seven by 13%. Concerning complementary feeding, there was varied age of introduction of complementary feeding. More than half (54.3%) of the caregivers did not adhere to the WHO and MOH of Uganda guidelines of introducing complementary feeding at 6 months while less than half (45.7%) adhered to the guideline. In addition, the study found that an equal proportion of the children (50:50) were still breastfeeding and had been weaned. Own‐grown foods were 22 times more consumed in the studied households than the purchased foods and the sale of agricultural produce contributed three‐quarters of the main source of income in the studied households compared to other sources of income such as formal employment and causal labor that altogether contributed to only one‐quarter of the total household income.

**TABLE 2:** Sociodemographic characteristics of the caregivers.

Variables	*n*	%
Sex of the caregiver		
Male	29	25
Female	87	75
Age of the caregiver (years)		
>18	2	1.7
18–25	41	35.3
26–35	36	31
36–45	29	25
46+	8	7
Marital status		
Single	7	6
Married	88	75.9
Separated	12	10.3
Widowed	9	7.8
Occupation household head		
Formal employment	8	6.9
Causal laborer	11	9.5
Farmer	87	75
Others	10	8.6
Occupation caregiver		
Formal employment	3	2.7
Causal laborer	14	12
Farmer	87	75
Others	12	10.3
Education level household head		
Education level of the caregiver		
No formal education	13	11.2
Primary	66	57
Secondary	35	30.1
Tertiary	2	1.7
Household size		
>7 people	54	46.5
<7 people	62	53.5
Age of the children		
Children aged below 6 months	38	32.8
Children aged 7–12 months	49	42.2
Children aged 13–23 months	29	25
Age porridge introduced		
<6 months	28	24.1
6 months	53	45.7
>6 months	35	30.2
How food is obtained		
Farming	111	95.7
Purchase	5	4.3
Who is responsible for providing for the household		
Family head	55	47.4
Wife/mother	40	34.5
Both husband and wife	17	14.7
Grandparent	4	3.4
Decides family income use		
Family head	66	56.9
Wife/mother	36	31
Both husband and wife	11	9.5
Grandparent	3	2.6
Who is responsible for cooking and home/child care		
Family head	1	0.9
Wife/mother	109	94
Both husband and wife	4	3.4
Grandparent	2	1.7
The main source of family income		
Sale of agricultural produce	87	75
Others (causal labor and formal employment)	29	25
Where the caregiver gave birth		
Hospital	82	70.7
Home	33	28.4
Prison	1	0.9
Sex of the child		
Female	58	50
Male	58	50
Childbirth weight		
<2.5	9	7.8
≥2.5	90	77.6
Do not know	17	14.6
Child breastfeeding		
Yes	58	50
No	58	50

*Note*: Data were based on a sample size of 116 caregivers. A caregiver is the mother/father of the child or legal guardian who takes care of the child in the absence of the mother/father.

Concerning gender roles and responsibilities, the male family head was 13 times more responsible for the provision of the household needs than the female caregiver and had 33 times more than the shared responsibility between the wife and the husband. Additionally, the male family head was two times more responsible for the decision‐making on family income usage than the female caregivers and had six times more than the shared responsibility between the wife and the husband. In terms of feeding and child care, women largely (94%) decided on food to cook and cared for the children. The current study revealed that more than 42% of the caregivers gave birth in the medical facility than at home. The birth records indicated that more than three‐quarters (77.6%) of children had normal birth weights and among the studied children, there was an equal proportion of boys to girls.

### 3.2 Food safety knowledge of the caregivers

**FIGURE 1:**
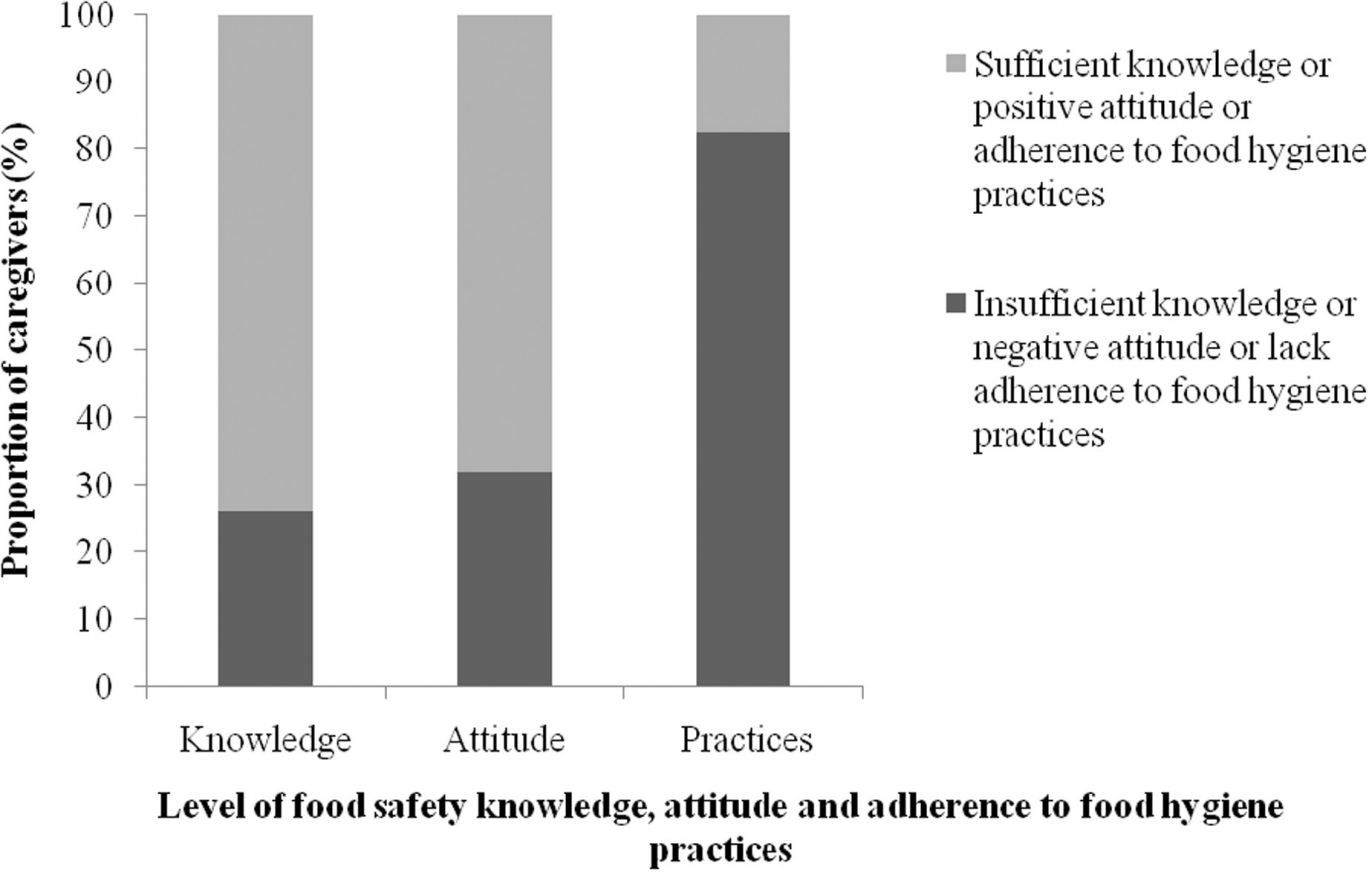
Distribution of food safety knowledge, attitude, and level of adherence to food hygiene practices among caregivers based on a sample size of 116 caregivers. A caregiver is the mother/father of the child or legal guardian who takes care of the child in the absence of the mother/father.

Figure 1 shows the overall distribution scores of food safety knowledge, attitude, and level of adherence to food hygiene practices of the caregivers. There was a high proportion of caregivers with sufficient food safety knowledge (74.1%) and positive attitude (68.1%) although caregivers' adherence to food hygiene practices was low by factors of 4 and 3.8, respectively.

Table 3 indicates that the largest proportion (93.1%) of the caregivers were aware of the importance of ensuring food safety, and above average (60.3%) of them could correctly define safe food. This perceived benefit is vital in ensuring food safety at the household level. Eight of ten of the caregivers perceived agrochemical residues and microorganisms as the greatest threat to food safety. All (100%) of the caregivers were unable to recognize mycotoxin, allergens, veterinary drug residues, heavy metals, unsafe packaging materials, food chemical issues (e.g., hormones and food additives), health issues (e.g., food nutrient imbalance), and food regulatory issues (e.g., food inspection and labels) as a threat to food safety. Therefore, food safety training should be focused on threats that are less known to rural communities. There are many ways by which one can acquire food safety knowledge. In this study, the largest proportion (38%) of the caregivers acquired food safety knowledge through formal food safety training in health facilities, especially during antenatal care. Nearly equal proportions (20%, 22%, and 20% respectively) of the caregivers acquired food safety from schools where caregivers attended formal education, from friends and relatives, and from radio programs. These means of knowledge acquisition mode should be sustainably strengthened.

**TABLE 3:** Food safety knowledge of the caregivers.

Questions	The proportion of households with response to specific knowledge aspect	
	(*n*)	(%)
Never participated in a food safety training		
Yes	51	44
No	65	56
Source of information regarding food safety		
Doctor/health work	19	38
Friends and relatives	11	22
Radio	10	20
School	10	20
Basic food hygiene practices		
Washing hands with soap	4	3.4
Wash baby's hands	1	0.9
Cover food well	7	6
Clean utensils	4	3.4
Keep flour in a clean dry place	3	2.6
Ensure a clean working environment	1	2.6
Do not know	96	82.8
Importance of preparing a safe food		
Protect children from foodborne diseases	108	93.1
Do not know	8	6.9
Definition of safe food		
Nutritious food	33	28.4
Food free from contaminants	70	60.3
Well‐covered food	4	3.4
Well‐cooked food	5	4.3
Hot food	3	2.6
No response	1	0.9
The perceived greatest threat to food safety		
Chemical and chemical residues	58	50
Microorganisms and their metabolites	37	31.9
Physical elements, for example, sand and metal pieces	17	14.7
Do not know	4	3.4
Symptoms of foodborne illnesses		
Vomiting	12	10.3
Stomach upset	50	43.1
Diarrhea	54	46.6
The child never suffered from symptoms of foodborne illnesses when introduced to the porridge		
Yes	72	62.1
No	44	37.9
Things that are done to make food unsafe		
Not washing hands with soap	4	4.3
Not covering food well	7	6
Unclean utensils	4	3.4
Keep flour in an unclean damp place	3	2.6
Unclean working environment	1	0.9
Nothing	96	82.8
Not aware of food safety authority		
Yes	54	46.5
No	62	53.5
Do not know the roles of food safety authority		
Yes	68	58.6
No	48	41.4

*Note*: Data were based on a sample size of 116 caregivers. A caregiver is the mother/father of the child or legal guardian who takes care of the child in the absence of the mother/father.

Eight of ten of the respondents stated stomach upset and diarrhea as symptoms of foodborne illness. None of the caregivers mentioned paralysis, fatigue, and dizziness as symptoms of foodborne illness. In addition, although more than half (53.5%) of the caregivers were aware of the food safety authority, 17% more of the caregivers did not know their roles in ensuring food safety.

### 3.3 Caregivers' food safety attitude

The food safety attitude of the caregivers is summarized in Table 4, indicating nearly all (97.4%) of the caregivers have shown a positive attitude concerning most food safety aspects. For instance, 9 of 10 caregivers had a positive attitude toward the safety of homegrown agricultural crop commodities while 2 of 5 of the caregivers had a negative attitude toward the safety of purchased raw materials used for making malted millet–sesame–soy composite. However, nearly three‐quarters (70.7%) were willing to pay the extra price for safe raw materials for making malted millet–sesame–soy composite.

**TABLE 4:** Attitude toward food safety.

Questions	The proportion of households with response to specific attitude aspects	
	(*n*)	(%)
Food safety is not an important issue at home (food safety benefits)		
Disagree	113	97.4
Neither agree nor disagree	1	0.9
Agree	2	1.7
Have confidence in the food safety authority		
Disagree	18	15.5
Neither agree nor disagree	27	23.3
Agree	71	61.2
Locally made malted millet–sesame–soy composite porridge is not a safe product		
Disagree	95	81.9
Neither agree nor disagree	9	7.8
Agree	12	10.3
Homegrown raw materials used for making malted millet–sesame–soy composite are safe		
Disagree	7	6
Neither agree nor disagree	8	6.9
Agree	101	87.1
Not willing to pay the extra price for safe raw materials for making malted millet–sesame–soy composite		
Disagree	82	70.7
Neither agree nor disagree	10	8.6
Agree	24	20.7
Raw materials purchased for making malted millet–sesame–soy composite are safe		
Disagree	45	38.8
Neither agree nor disagree	11	9.5
Agree	60	51.7

*Note*: Data were based on a sample size of 116 caregivers. A caregiver is the mother/father of the child or legal guardian who takes care of the child in the absence of the mother/father.

### 3.4 Food safety self‐reported practices

**FIGURE 2:**
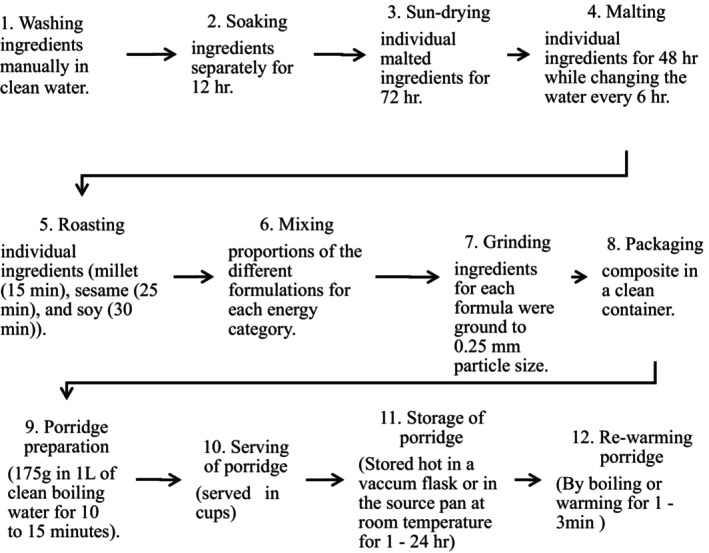
Detailed porridge production process. The porridge was made from three ingredients: millet (*Eleusine coracana*), sesame (*Sesamum indicum*), and soybeans (*Glycine max*).

Food safety practices of caregivers of children aged 6 to 23 months are recapitulated in Table 5. Generally, most caregivers embraced general hygiene practices such as the use of clean water sources (borehole and protected well) (74.1%), use of pit latrines (98.3%), and washing hands with water and soap after visiting the pit latrine (84.5%). However, 7–9 in 10 households did not adhere to specific areas of food hygiene such as hand washing before preparing food and feeding the child. Concerning storage of the locally made complementary food composite, majority (93.1%) of caregivers stored processed composite flour for 2 to 14 days, and only 2 of 116 caregivers stored for more than 30 days. Nearly half (47%) of the households cooked the composite for an estimated time of 15 to 30 min. Only 15 of 116 caregivers could not estimate the time taken to cook the composite (See Figure 2 for a detailed porridge production process). Meanwhile, three‐quarters of the respondents stored the cooked porridge at ambient temperature (typically 25 to 30°C) and used it within a period of 8 h. Regarding utilization of the stored porridge, majority (76.7%) did not warm, and largely (61.2%) were stored in vacuum flasks. Those who do not use vacuum flasks reheated before taking by boiling for 10 to 15 min and reheated without reaching boiling for 2 to 3 min.

**TABLE 5:** Self‐reported food safety practices of the caregivers.

Questions	Practices of the caregivers	
	(*n*)	(%)
Source of clean water		
1. Borehole	58	50
2. Protected well	28	24.1
3. Well	30	25.9
Do you have a pit latrine?		
1. Yes	114	98.3
2. No	2	1.7
What is done after visiting the pit latrine?		
1. Hand washing	98	84.5
2. Nothing	18	15.5
What is done before preparing food?		
1. Hand washing	8	6.9
2. Nothing	108	93.1
What is done before feeding a child?		
1. Hand washing	33	28.4
2. Nothing (“Just get food and feed the child”)	83	71.6
Household members treated when suffering from foodborne illness		
Yes	32	72.7
No	12	27.3
If yes, treated from where?		
Medical unit	22	52.4
Home (self‐medication, both conventional and traditional)	20	47.6
How household produce is dried		
Spreading on the ground	18	15.5
Spreading on the mat/tarpaulin	91	78.4
Spreading on polythene/“kavera”	4	3.4
Spreading in the tray	2	1.7
No response	1	0.9
How raw materials are stored		
Sack	47	40.5
Sack and raised ground	12	10.3
Polythene bag (kavera)	8	6.9
Polythene bag (kavera) and raised ground	2	1.7
Closed container/bucket	16	13.8
In a dry place	4	3.4
In the bags	1	0.9
N/A	1	0.9
No response	8	6.9
Do not know	17	14.7
Where raw materials are stored		
House/grass thatched	107	92.2
Store	3	2.6
Granary	6	5.2
No. of days the MMSSC is stored		
2–7 days	73	62.9
8–14 days	35	30.2
15–30 days	5	4.3
>30 days	3	2.6
How long the porridge is cooked		
10–15 min	22	18.9
>15–30 min	54	46.6
>30–60 min	20	17.2
>60–90 min	1	0.9
>90–120 min	4	3.4
Do not know	15	12.9
Stores cooked porridge		
Yes	86	74.1
No	30	25.9
Hours cooked porridge is stored		
1–4 h	36	42.4
5–8 h	29	34.1
9–12 h	20	23.5
How did the respondents store the cooked porridge?		
Covered cooking pan	7	6
Vacuum flask	71	61.2
Covered in a jug	9	7.8
N/A	29	25
How the respondent warms the cooked porridge		
Boil	6	5.2
Warm	14	12.1
Do not warm it	89	76.7
N/A	7	6

*Note*: Data were based on a sample size of 116 caregivers. A caregiver is a mother/father of the child or a legal guardian who takes care of the child in the absence of the mother/father.

### 3.5 Association of caregivers' characteristics with food safety knowledge and attitude

Multivariate binary logistic regression analysis in Table 6 indicated that gender role in decision‐making on the household income (*p* = .034), frequency of food safety training (*p* = .041), households with children who had ever suffered from foodborne illness (*p* = .001), and household with children aged 1 to 17 years (*p* = .002) were significantly associated with food safety knowledge of the caregivers. Households with the male gender taking the lead in decision‐making on household income were associated with nearly 2 (1.831)‐fold increase in the level of food safety knowledge. Similarly, caregivers who frequently attend food safety training were more than half (0.602) better in food safety knowledge than their counterparts who only attend once. Furthermore, households with children who have ever suffered from foodborne illness were nearly 1 (0.852)‐fold better in food safety knowledge than those who have never. Also, households with older children aged 1 to 17 years have almost more than half (0.58)‐fold increase in food safety knowledge compared to those who have only younger children aged under 5 years.

**TABLE 6:** Association of caregivers' characteristics with food safety knowledge and attitude.

Variables	Knowledge			Attitude		
	*β*	SE	*p*‐value	*β*	SE	*p*‐value
Residence of caregiver	1.049	.610	.086			
Sex of the caregiver	.691	.893	.439	−.251	.642	.695
Age of the caregiver	−.036	.033	.274	−.037	.026	.153
Occupation of household head	−1.609	.960	.094			
Occupation of the caregiver	1.001	.900	.266	−.767	.653	.240
Marital status	1.251	1.108	.259	.010	.749	.989
The main source of income	−.904	.901	.316	.889	.610	.145
Decision on household income	1.831	.865	.034*	1.925	.706	.006*
Decision on food to cook	−2.453	1.622	.130			
Responsibility for the household provision	−.999	.755	.186	−1.128	.724	.119
Trained in food safety	−.318	.990	.748	−.578	.797	.468
Frequency of food safety training	.602	.295	.041*	.356	.202	.079
Children in the household ever suffered from foodborne illness	.852	.266	. 001*	−.111	.166	.504
Where the child was treated	−.168	.902	.852			
Children in the household age 1–17 years	.583	.192	.002*	.254	.124	.041*
Children in the household age ≤5 years	.342	.379	.368			
Children in the household age ≤2 years	.029	.419	.946	−.148	.348	.672
Constant	1.173	2.759	.671	1.048	1.586	.509

*Note*: A caregiver is the mother/father of the child or legal guardian who takes care of the child in the absence of the mother/father.

Abbreviations: *β*, regression coefficients; SE, standard error.

* indicates the variables that significantly predicted food safety knowledge and food safety attitude.

Regarding attitude, binary multivariate logistic regression analysis indicated that both genders taking the lead in decision‐making on household income and households with children aged 1 to 17 years were significantly associated with a positive attitude toward food safety (*p* = .006 and *p* = .041), respectively. Households with the male gender taking the lead in decision‐making on household income are associated with a virtually two (1.925)‐fold decrease in a positive attitude toward food safety. In addition, households with children aged 1 to 17 years are associated with about a quarter (0.254)‐fold increase in a positive attitude toward food safety. Generally, both decision‐making on household income and households with children aged 1 to 17 years significantly predicted FSK and FSA, which showed a significant positive correlation (*r* = .406, *p* = .000).

### 3.6 Common themes generated from FGD based on the health belief model

The summary of common themes generated from FGD and caregivers' quotes is shown in Table 7. Common themes are reported below.

**TABLE 7:** Quotes from FGD based on health belief constructs.

Common themes based on health belief constructs	Quotes from focus group discussions
*Perceived susceptibility*	
The susceptible group in the household	“Anybody in the community is susceptible to food‐borne illness. But children especially those from six months (on the introduction of complementary foods) to 23 months.”
*Perceived severity*	
Gastrointestinal disturbances	“Adults mostly suffer stomach ache and diarrhea. But children suffer from vomiting, loss of appetite, and fever in addition to stomach ache and diarrhea.”
How long the condition lasted?	“The time lag varies, from 1 to 14 days but rare cases last for more than 14 days.”
Medical treatment	“Many of those who get sick seek medical attention due to the severity of the illness. Children especially were taken to hospitals or medical clinics. Adults and children with the less severe case do self‐medication from home or get healed without medication.”
*Perceived benefits*	
Importance of preparing safe food	“Prevent children from food‐borne illnesses”
Identify safe food practices that prevent foodborne illness	Hand washing with clean water and soap after visiting the pit latrine, before preparing food, and before feeding the child Know the time taken to cook porridge, best way to rewarm porridge
*Perceived barrier*	
Hygiene practice (inadequate pit latrine)	“More than half of the community share pit latrine. This makes cleaning and provision of water and soap for washing hands after use very challenging.”
The challenge of drying agricultural produce	“Most of us dry on the mat, polyethylene bags (‘Kavera’), and tarpaulin because those who do not have them can borrow them. But those who do not have and cannot borrow do dry their agricultural produce on bare ground.”
Time and caregiver's workload	“We (women) are the ones responsible for household care mostly obtaining and preparing food, household hygiene in addition to children's care. This work is overwhelming and makes us take shortcuts, especially in food preparation and child feeding.”
*Self‐efficacy*	
Confidence	“We always get food safety knowledge when we go for antenatal visits in hospitals and health centers” “Locally made malted‐millet‐sesame‐soy composite porridge is a safe product for our children aged 6 to 23 months.” “Both homes grown and purchased raw materials used for making malted millet sesame soy composite are safe.” “Most of us are willing to pay the extra price for safe raw materials for making malted‐millet‐sesame‐soy composite. I rather spend money on a good thing that will prevent diseases.” “I have confidence in food safety authority, especially in meat inspection but not other produce.”
Food‐handling control	“Only what you have produced is under your control but one has to decide to either purchase expensive good quality produce or cheap poor‐quality produce. I go for cheap products because high‐quality products are very expensive, some of us cannot afford them.” “Most of us prepare baby's porridge once a day and it is consumed for the whole day.”
Food safety handling of leftover	“The leftover is either put in a well‐covered flask, Jug, or saucepan. The one left in the Jug or saucepan will then be re‐warmed, boiled, or given to the child cold depending on the time available.”
False confidence	Nutritious food is a safe food
*Cues to action*	
Audio‐visual catching message	“Posters in hospitals and clinics showing a person with diarrhea, vomiting as a result of consuming unsafe food or not following proper food hygiene practices.” “Scary media report on television and radios highlighting socio‐economic losses resulting from unsafe foods.”
Shocking message	“Experts talk shows on radios and televisions educating the public on the dangers of consuming contaminated foods.”

*Note*: The information was generated from a total of eight focus groups consisting of 6 to 10 caregivers. A caregiver is the mother/father of the child or legal guardian who takes care of the child in the absence of the mother/father.

#### 3.6.1 Perceived susceptibility and severity

Caregivers believed that children were more susceptible to foodborne illness than other household members. They also believed that their household members have ever suffered from symptoms of foodborne illness such as vomiting, stomach ache, and diarrhea, resulting from consuming contaminated food. These conditions were reported to last from 1 day to more than 14 days in some cases. Among the children who had the above symptoms, the majority were treated and others got healed without treatment. Commonly, treatment was obtained from medical facilities, but others did self‐medication from home using both conventional and traditional medicine.

#### 3.6.2 Perceived barriers

Inadequate sanitation facilities such as pit latrines and drying equipment, especially tarpaulin/polyethylene bags, were reported as barriers to food safety. Whereby, the community believed that more than half of the households share pit latrines, and also some dry their agricultural produce on bare ground. Additionally, women's workload was considered a big challenge and caregivers reported that they get exhausted and take shortcuts to prepare food and feed the hungry crying children. Furthermore, caregivers reported inadequate knowledge to prepare children's food, for instance, the time needed to safely cook porridge was unknown. They just prepare according to convenience. The caregivers stated, “When the bubbles in the porridge clear and a good aroma start, I will know the porridge is ready. But sometimes, when the child starts crying nobody can wait for the above features.”

#### 3.6.3 Self‐efficacy

High self‐efficacy in food safety practices was observed in the FGDs. Group members reported that most of the caregivers have safe water sources or share a pit latrine. They also believed that only a few households do not use a pit latrine. Community discussions indicated that virtually all the caretakers wash their hands with soap and water after visiting the pit latrine and only a small portion do not. Additionally, the community perceived that the majority of the caregivers were willing to pay an extra price for safe raw materials for making malted millet–sesame–soy composite and they believed that food safety is an important issue at home. But some group members did not know basic food hygiene. Although the FGD members knew the roles of food safety authority, they had no confidence in food safety authority, especially in the regulation of cereals safety.

#### 3.6.4 Cues to action

The FGDs revealed that important messages concerning food safety knowledge and practices can be passed from generation to generation from parents/caregivers to the children in addition to acquiring it formally right from schools and medical facilities where the caregivers attend antenatal care. Posters in hospitals and clinics showing a person(s) with diarrhea and vomiting as a result of consuming unsafe food or not following proper food hygiene practices need to be put appropriately in medical centers where community members usually go to seek medical attention. Poster pictures should be brightly colored and information translated into the local language. Additionally, the FGDs members perceived scary media reports on television and radio on the health and economic impact of food contamination and experts' talk shows on the dangers of consuming contaminated foods to trigger recommended food safety actions.

## 4 DISCUSSION

### 4.1 Sociodemographic characteristics of the caregivers

In terms of complementary feeding, the study indicated that less than half (45.7%) of the caregivers introduce complementary feeding at 6 months as recommended by the World Health Organization (WHO, 2003) and the Ministry of Health (2009). However, the remaining proportion of caregivers introduced complementary foods either before or after 6 months. Similar to the finding of this study, Aber et al. (2018) noted low adherence to complementary feeding guidelines in a rural setting in Uganda. As a result, there is a need to discourage practices that hinder adherence to recommended practices because, before 6 months of age, the child's digestive and immune systems are still underdeveloped to digest complimentary food and overcome any food‐related illnesses (Rao et al., 2011). Also, the late introduction of complementary foods after 6 months is equally inappropriate because of the energy gap since breast milk alone even from a well‐nourished mother cannot support a child's optimal growth. This is because the metabolizing energy intake of breast milk is 2.2–2.4 MJ/day and the mean energy requirement for babies at 6 months is approximately 2.6–2.7 MJ/day (632–649 Kcal/day) (Reilly & Wells, 2005). However, Prescott et al. (2008) argued that there is a growing need to revise the age of introduction of complementary food to 4 months due to various needs while maintaining breastfeeding to a minimum of 6 months. But as pointed out by Prescott and Tang (2005), there is a conflict between the introduction of complementary feeding from 4 to 6 months by some allergy prevention guidelines being practiced in Australia and the WHO recommendation of the introduction of complementary feeding until 6 months. These conflicting ideas need to be resolved through carrying out extensive research to harmonize the age of introduction of complementary feeding to realize good nutritional outcomes from complementary feeding. The current study revealed that nearly all (957%) of the consumed food in the studied household was own grown; only a small (4.3%) portion is purchased. The finding suggests that the foods consumed by this community are relatively safe since this study further revealed that the studied community perceived that their grown produce is relatively safe compared to what is purchased. This may apply to households who are economically constrained and might not afford relatively safe quality agricultural products sold in the market. Additionally, the implication of this is that, in case of crop failure, this community may suffer food insecurity coupled with the utilization of unsafe food. In agreement with our findings, other studies revealed that generally rural areas in low‐ and middle‐income countries have extensively been depending on their grown produce for household consumption (Adeyeye et al., 2022; Ejeromedoghene et al., 2020; Gebre & Rahut, 2021). This scenario is positive because the current study revealed that poor households can only be sure of the food quality and safety when it is own grown. Therefore, the government should strengthen the capacity of households to grow their food and diversify their livelihood activities to enhance resilience in case of crop failure.

Concerning gender roles and responsibilities, caregivers reported that in nearly half (47.4%) of the male‐headed households, decision‐making on income usage and responsibility for the provision of the household needs were carried out by the male gender. This could have been because the female gender earns low income due to their unpaid care and domestic work. (Guloba et al., 2018). Largely women (94%) only decided on food acquisition and preparation. This was classical of rural African households where women chiefly make the decision only on food since they are gatekeepers of food and nutrition security. And, mainly men decide on household income. Gender roles and responsibility have changed over time. Initially, in an African setting, specifically in northern Uganda, the husband/family head was responsible for all the household decision‐making and provision of a decent household standard of living. Similarly, in Tanzania, Anderson et al. (2017) found that men held more power in terms of household decision‐making and provision except in specialized areas. This may have negative implications on household food safety and food and nutrition security as a whole in case of crop failure since the gender that makes the critical decision on household food consumption may not be fully involved in the decision on household income usage. Thus, decisions on household income need to be made collectively to ensure food safety and food and nutrition security as a whole.

### 4.2 Food safety knowledge of the caregivers

In terms of knowledge, all of the caregivers knew the importance of preparing safe food to prevent foodborne illnesses. However, the majority (82.4%) did adhere to food hygiene practices such as washing hands with soap and clean water before preparing and eating food or feeding the child, ensuring a clean working environment, cleaning utensils, covering food well to avoid cross‐contamination, and keeping food in a clean dry place. This could partly be explained by the low level of education attained by the caregivers (Table 2). A similar scenario was reported by Webb and Morancie (2015) whereby most (81%) of the participants knew the importance of hygiene practice such as proper hand washing but only 2% practiced it well. This reflected weakness could be due to time constraints and inadequate resources needed to perform the task as reported by Brannon et al. (2009). However, washing hands with antibacterial soap eliminates >95% of total coliforms even for a shorter exposure time (Toshima et al., 2001). The barriers to good food hygiene practices thus need to be addressed and social‐demographic factors such as frequent food safety training should be encouraged to enhance knowledge. However, Ncube et al. (2020) acknowledged that the hygiene practices such as hand washing need validation by observation because a significant deviation in the observation checklist from the reported practice was discovered, whereas da Cunha et al. (2019) affirmed that observed practice is directly influenced by knowledge and attitude and is closer to actual practice. Differing from the study findings, Kunadu et al. (2016) found that most of the participants in their study reported washing their hands with antibacterial soap after visiting a toilet and before food preparation to prevent foodborne illnesses. This deviation could be due to the formal environment (restaurant) where their study was conducted unlike the typically rural households considered in this study. This study showed that although more than half of the caregivers were aware of the food safety authority, the same proportion of the caregivers did not know their roles the authority in ensuring food safety. Policymakers, therefore, need to reinforce food safety departments at all levels and they should be made functional.

### 4.3 Caregivers' food safety attitude

Overall, almost all (97.4%) caregivers had a positive attitude toward food safety as an important concern at home with more than half (61%) of them having confidence in the food safety authority. The caregivers had a positive attitude toward the safety of both the homegrown and purchased raw materials used for making malted millet–sesame–soy composite (87% and 51.7%, respectively). Although the FGDs pointed out that “as long as you have money, you can always buy a good quality safe food, sometimes even better than own grown.” This statement was attested by Safari et al. (2017), who found out that food safety attitudes can be influenced not only culturally or socially but also economically such as food prices. Affirming the aforementioned studies, this study found that nearly three‐quarters (70.7%) of the caregivers were willing to pay the extra price for safe raw materials for making malted millet–sesame–soy composite. Only less than a quarter of the caregivers were not willing because of financial constraints. More than a third of the caregivers had a negative attitude toward the safety of purchased raw materials used for making malted millet–sesame–soy composite. This could be because quality raw materials are usually highly priced compared to poor‐quality ones. But, the willingness to pay the extra price is dependent on the economic situation of the household. Therefore, households need to be economically empowered to utilize safe foods. The finding from this study replicated Meysenburg et al.'s (2014) findings which indicated that food handlers reported that “the only thing you can control is what you have in your house.” The caregiver's positive attitude needs to be translated into good food hygiene practices by breaking the barriers such as economic constraints and the caregiver's workload and addressing social economic factors such as the decision on household income that influence attitude. The male gender's decision on household income positively influences attitude. This could be because they own more resources and earn more income. The female gender thus needs to be empowered since they are doorkeepers of food safety as revealed by this study.

### 4.4 Food safety self‐reported practices

In terms of postharvest handling, the majority of caregivers (83.6%) did not dry their agricultural produce on the bare ground since they had personal or shared/borrowed tarpaulin for drying agricultural produce. Tarpaulin ownership was limited because they are quite expensive for a rural household setting. After drying agricultural products, most (89.7%) of the households did not store their produce on raised ground predisposing them to mold growth, rodents, and insect infestations that compromise food quality and safety. Only 10.3% of households stored the produce on raised ground. Thus, there is a need to sensitize the community about the danger of improper postharvest management and training on best practices. Almost all (92.2%) of the agricultural produce was stored by stockpiling on polypropylene sacks and baskets in grass‐thatched houses that were used for sleeping by the household members due to fear of thieves as reported by the community. This practice should be discouraged because it predisposes the produce to cross‐contamination, so the local authority in rural communities should ensure = social security. Our finding is comparable to that of Viola (2017), who found three‐quarters of the products are stored in rooms at home after harvesting for fear of thieves. The caregivers revealed that only 2.6% of the households stored their agricultural produce in the local community store. Community storage of the product should be encouraged because the facility offers better services that reduce contamination, thus ensuring food quality and safety. More than half (62.9%) of the caregivers stored processed composite flour for feeding children aged 6 to 23 months for 2 to 7 days. The caregivers reported that they were trained to keep the composite for a short duration to prevent the proliferation of spoilage agents. The rest of the caregivers (34.5%) stored for 8 to 30 days and only very few (2.6%) caregivers stored for more than 30 days. These varying storage days are, however, challenging in case they need to access a wider market. This means irrespective of the source of raw materials, the exact shelf‐life needs to be determined.

Concerning time and temperature control, nearly half (46.6%) of the caregivers took an estimated 15 to 30 min to cook MMSSC (the locally made complementary food) and only 12.6% did not know the time taken to cook the composite. In this rural setting, cooking is done arbitrarily. The cooked porridge is usually stored for a range of 1 to 24 h. The stored porridge is usually rewarmed by boiling (5.2%) and warming (12.1%) for a very few (2–3) minutes before taking it. Warming by boiling should be encouraged because, in case of any germination of spores, the vegetative form can be destroyed by boiling. Although in case of toxin production resulting from contamination at preharvest and postharvest, mostly during improper storage of raw materials and flour, simple boiling may not eliminate hazards. Therefore, proper pre‐ and postharvest management should be encouraged. Additionally, porridge should be properly stored to eliminate hazards. Warming for very few (2–3) minutes, however, should be discouraged as it does not destroy the vegetative form of the microorganism but provide a conducive environment for their proliferation. A greater percentage (76.7%) of the caregivers do not warm the leftover porridge because they store the leftover porridge in a vacuum flask to keep it hot until use. Several studies have reported an enormous challenge of temperature and time control to many food handlers (Baş et al., 2006; Buccheri et al., 2007; Ncube et al., 2020; Walker et al., 2003; Webb & Morancie, 2015).

### 4.5 Association of caregivers' characteristics with food safety knowledge and attitude

In this study, results from binary multivariate logistic regression models substantiated that many of the sociodemographic characteristics could not give a significant association between sufficient food safety knowledge and a positive attitude toward food safety. However, gender role in decision‐making on household income was a significant predictor for both sufficient knowledge and positive attitude. The model further revealed that households with the male gender taking the lead in decision‐making on household income were associated with twofold increase in the level of food safety knowledge, which could have been because men earn more income than women and so enabling them to support food safety programs could enhance the household's food safety knowledge (Guloba et al., 2018). But since the study revealed that mainly women make a decision on food acquisition and preparation, they need to be empowered and participate equally in household income decision‐making. Conversely, households with the male gender taking the lead in decision‐making on household income are associated with a virtually twofold decrease in a positive attitude toward food safety. The decrease in positive attitude could have been because most caregivers play a lesser role in household income and so they cannot decide on appropriate food safety measures that require finances. Furthermore, the frequency of food safety training and households with children who have ever suffered from foodborne illness significantly predicted sufficient food safety knowledge. Whereby, caregivers who frequently attended food safety training had more than half an increase in food safety knowledge than their counterparts who only attend once. Additionally, households with children who had ever suffered from foodborne illness had a nearly onefold increase in food safety knowledge than those who had never. Equally, households with children aged 1 to 17 years had significantly sufficient food safety knowledge and positive attitude with more than half (0.58)‐fold increase in food safety knowledge compared to those who had only younger children aged under 5 years. Also, a quarter (0.254)‐fold increase in a positive attitude toward food safety was observed in a household with children aged 1 to 17 years. The above predictors could plausibly be explained by the experience of food safety that a caregiver gained during training and over the years of childbearing which build their self‐efficacy and positive attitude (Meysenburg et al., 2014). Nee and Sani (2011) and Abdul‐Mutalib et al. (2012), however, found deviant results, indicating an insignificant correlation between experience in food‐handling operation and increased food safety knowledge and attitude. In this study, however, a significant positive correlation between food safety knowledge and attitude was observed (*r* = .406, *p* = .000). Several studies have revealed that good predictors of safe food‐handling practices are sufficient food safety knowledge and a positive attitude (Abdul‐Mutalib et al., 2012; Kunadu et al., 2016; Ncube et al., 2020). However, according to Kunadu et al. (2016), Nassanga et al. (2018), and Ncube et al. (2020), knowledge may not necessarily be translated into appropriate practices, and self‐reported practices may not be the actual practice. Therefore, self‐reported practices need to be verified with an observation checklist.

### 4.6 Themes generated from FGD based on the health belief model

#### 4.6.1 Perceived susceptibility and severity

FGDs based on health belief model constructs as shown in Table 7 revealed that children were more susceptible to foodborne illness compared to other age groups in the household possibly due to their underdeveloped immune systems (Agostoni et al., 2009; Du Plessis et al., 2013). FGDs further revealed that adults generally suffered stomach aches and diarrhea but children suffered vomiting, loss of appetite, and fever in addition to stomach aches and diarrhea. These findings are strongly supported by a previous study by Meysenburg et al. (2014), indicating that children and the elderly were more susceptible to foodborne illness due to their weaker immune systems. The caregivers believed that their household members have ever suffered from symptoms such as vomiting, stomach ache, and diarrhea resulting from consuming contaminated food. These conditions were reported to last from 1 day to more than 14 days in some cases. This implied that the caregivers knew some of the consequences of eating contaminated foods. However, the study findings presented in Table 3 indicate that the caregivers had a low perception of susceptibility and severity to foodborne illnesses. This could have resulted from inadequate and late diagnosis of foodborne illnesses and lack of experience with foodborne illnesses. Another reason includes ignorance of the serious health consequences of food contamination such as stunting in children and a more life‐threatening condition such as liver cancer and hemolytic uremic syndrome (Buzby, 2001; Meysenburg et al., 2014). Additionally, this could have resulted from the respondents' failure to recognize that they are susceptible to foodborne illness and the high degree of severity of infection. Caregivers' low perception of their susceptibility to FBI and its severity have been a result of not acknowledging the reality due to fear of undesired outcomes. Thus, this opportunistic bias may lead to invalid conclusions and this can be overcome by rigorous methods of data collection which may be costly (DeCoster et al., 2015). Among the children who had the above symptoms, the majority (72.7%) were treated and 27.3% got healed without treatment. Treatment was obtained from the medical unit (52.4%) and others (47.6%) used self‐medication.

In this particular study, caregivers did not understand the risks of food contamination posed by unhealthy food handlers. Similar findings by Osaili et al. (2011) showed that food handlers lacked adequate knowledge of the risks posed by unhealthy primary food preparers. Although factors such as food contamination due to poor agricultural practice and cross‐food contamination from the food handlers suffering from diseases such as diarrhea, cough, and flu have been reported to increase the risk of foodborne illnesses (da Cunha et al., 2014; McIntyre et al., 2013; Ncube et al., 2020; Pichler et al., 2014), food safety training should focus on the threats that are unknown to caregivers.

#### 4.6.2 Perceived benefits

Caregivers acknowledged that the overall goal of safety practices is to prevent children and other household members from getting foodborne illnesses. The food safety practices pointed out by the members included: hand washing after visiting the latrine, before preparing food, and before feeding the child; drying agricultural produce on tarpaulin; cleaning utensils before preparing food, covering leftover food in a saucepan, jug, or flask; and rewarming leftover foods. Although these food safety practices were known by caregivers, many other food hygiene practices were not adhered to. These include hand washing with soap and clean water, washing hands before preparing food and feeding the child, not drying agricultural produce on the bare ground, storing the produce on raised ground, and proper warming of leftover food. These conflicting food safety practices are expected because according to the health belief model, the lower the perceived susceptibility and severity, the lesser the perceived benefits of taking action (food safety practices) and vice versa (Pourtaheri et al., 2018).

#### 4.6.3 Perceived barriers

In addition, caregivers believed barriers to practicing food safety include inadequate sanitation facilities such as pit latrines and drying equipment, especially Turpin/polyethylene bags. Concerning the identified barrier, more than half of the households share a pit latrine, and also some caregivers dry their agricultural produce on the bare ground. Furthermore, women's workload was considered a big challenge. Caregivers reported that the women get exhausted and take shortcuts to prepare food and feed the hungry crying children. This finding is comparable to that of Meysenburg et al. (2014) and not different from employers in a formal setting (restaurants) who fail to adhere to set food safety practices due to time constraints and limited resources (Webb & Morancie, 2015). Another barrier stated by the caregivers was inadequate knowledge to prepare children's food. For example, the time needed to cook a safe porridge, whereby they just prepare according to convenience, “When the bubbles in the porridge clear and the good aroma starts, I will know the porridge is ready. But sometimes when the child starts crying, nobody can wait for the above features.” In support of our findings, most participants in various studies did not know the critical temperatures and time requirement in the control of microorganisms (Baş et al., 2006; Buccheri et al., 2007; Cape et al., 2007; Walker et al., 2003; Webb & Morancie, 2015). In agreement with our findings, Brannon et al. (2009) found that a lack of adherence to food safety guidelines could be due to time constraints and limited resources, whereas Anon (2003) emphasizes that improper time and temperature control is one of the causes of foodborne diseases. To promote food safety within rural communities, there is a need for intervention to address the highlighted barriers to food hygiene practices.

#### 4.6.4 Self‐efficacy

High self‐efficacy refers to the ability of a person to competently execute the action to avert health threats such as foodborne illness (Rosenstock et al., 1988). Our study revealed caregivers' high self‐efficacy in food safety practices. For instance, most of the caregivers have a safe water source (74.1%) and have or share a pit latrine (98.3%). Virtually all (84.5%) of the caretakers voiced washing their hands with soap and water after visiting the pit latrine. Additionally, almost three‐quarters (70.7%) of the caregivers were willing to pay an extra price for safe raw materials for locally making malted millet–sesame–soy composite. This finding indicates that as long as a household is economically stable, it will be in a position to acquire safe food. But those who cannot afford costly safe food will always go for cheap unsafe food. This phenomenon was explained by Safari et al. (2017) as food safety knowledge, attitude, and practices can be influenced culturally, socially, and economically, such as food prices among others. This is parallel to the finding by Meysenburg et al. (2014), who indicated that “the only thing you can control is what you have in your house.”

#### 4.6.5 Cues to action

Some cue to action found in this study includes passing important messages concerning food safety knowledge and practices from generation to generation (parents/caregivers to the children), acquiring formally right from schools, and medical units where the mothers attend antenatal care. However, knowledge may be insufficient for behavior change, therefore, food safety programs should not only be designed to increase knowledge about food safety but also to emphasize the importance of adopting proper food‐handling practices. For these reasons, in our study, the caregivers pointed out the following as perceived triggers for the recommended food safety actions: posters in hospitals and clinics showing a person with diarrhea and vomiting as a result of consuming unsafe food or not following proper food hygiene practices needs. The posters should be put appropriately in medical units where expectant mothers and community members usually go to seek medical attention and the pictures should be brightly colored and the information translated into the local languages. Additionally, social marketing communication strategy with frightening media information about the dangers of consuming unsafe food should be aired out. This can be passed on radios and television, especially experts' talk shows on the dangers of producing and consuming contaminated foods.

The current study revealed that more than an average (60.3%) of the caregivers had sufficient knowledge of safe food. This can be explained by the fact that apart from formal training, the caregivers acquire knowledge informally. Caregivers reported acquiring knowledge from family and friends, radio programs, schools they attended, and health workers during antenatal care. However, nearly a third (28.4%) had false confidence in knowing that safe food is the same as nutritious food. Similar spurious confidence was reported by Meysenburg et al. (2014), whereby study respondents reported that safe food is determined by appearance or smell. But many (60.3) participants in this study affirmed that even if food is nutritious and looks and smells good, it may contain small organisms that cannot be seen.

The study found that an equal proportion (50%) of the caregivers perceived chemical and agrochemical residues as the greatest threat to food safety, followed by microorganisms (31.9%), and finally, extraneous physical elements (14.7%) such as sand and metal pieces. This finding deviates from those of Kunadu et al. (2016) who found that nearly all participants in their study reported microorganisms as the greatest threat to food safety. However, in both studies, participants did not recognize allergens, veterinary drug residues, heavy metals, unsafe packaging materials, food chemical issues (e.g., hormones and food additives), health issues (e.g., food nutrient imbalance), food regulatory issues (e.g., food inspection and labels), and microbial metabolites such as mycotoxin as the greatest threat to food safety. The authors in the current study argue that the divergent views could have resulted from participants' characteristics, the prior being from an informal rural household setting and the latter from formal food industrial setting. There are many ways by which one can acquire food safety knowledge; in this study more than half (56%) of the caregivers reported that they acquired knowledge from food safety training through nongovernmental organizations (NGOs), whereas the rest of the caregivers who had not attended training reported having acquired food safety knowledge from health workers, especially during antenatal care, friends, relatives, radio program, and schools where they attended classes. This finding is consistent with those of Trepka et al. (2006), Kwon et al. (2008), and Meysenburg et al. (2014), whose findings revealed that in addition to gaining experience in food‐related jobs, food safety knowledge can be acquired formally from school and informally from family. Thus, both the informal and formal means of knowledge acquisition should be strengthened to ensure community health and well‐being.

### 4.7 Study strengths and limitations

The study tool was pretested and validated. Additionally, data were collected by trained competent research assistants. Furthermore, the study was carried out among caregivers of children (6–23 months of age) in a rural setting. This contributed to the body of knowledge that was lacking in the area of food safety KAP in an informal rural domestic environment. The present study, however, had the following methodological constraints: we did not examine the KAP of the untrained group; only the caregivers who were trained on the use of locally made food formulae were recruited into the study; and since training has been associated with good knowledge and attitude, our findings may not be generalized to the entire community. Therefore, further research needs to be done to compare the KAP of trained and untrained caregivers. Additionally, the study design was cross‐sectional, therefore, it could not demonstrate a cause–effect relationship. Also, the purposive sampling method used is susceptible to sampling bias. Finally, the self‐reported practice technique of reporting food safety practices may result in inconsistency with the actual practice. Future research necessitates the use of an observation checklist to validate caregivers' food safety practices.

## 5 CONCLUSION

This study established that caregivers in the Acholi subregion of Uganda trained in the local production of malted millet–sesame–soy composite have sufficient food safety knowledge and a positive food safety attitude. However, most caregivers did not adhere to food hygiene practices.

The identified sociodemographic factors that hinder adherence to food safety practices included inadequate sanitation facilities such as pit latrines and drying equipment, especially Turpin/polyethylene bags. Furthermore, women's workload was considered a huge hindrance. Another barrier stated by the caregivers was inadequate knowledge to prepare children's food.

## 6 RECOMMENDATION

To address food safety issues at the rural domestic level where food is produced for both consumption and the market, food safety knowledge and attitude should be strengthened through delivering food safety education sustainably based on the health belief model. This will increase the perceived susceptibility and severity of the caregivers and other food handlers to foodborne illness while increasing their perceived benefits and reducing barriers to adhering to safe food‐handling practices. Results from this study highlighted the urgent need to strengthen medical centers to include food safety training during nutrition education training for mothers/caregivers attending antenatal care. The target participants in this setting are the doorkeepers of food safety according to the findings from this study. They will thus acquire food safety knowledge on proper household sanitation and hygiene, elimination of food contamination from garden to folk through good agronomic practices, good pre‐ and postharvest management, and proper food preparation taking into consideration time and temperature control. The government through the Uganda Nutrition Action Plan needs to enforce by‐laws on postharvest handling, especially abolishing drying produce on the bare ground. Women's workload of domestic and garden work should be addressed by conducting research that provides appropriate agricultural technologies that save time. Finally, laboratory analyses should be done on selected food commodities to ascertain the agricultural produce's safety and determine whether the food safety management systems are being practiced at household levels. This should be done alongside shelf‐life determination of the locally made complementary food to empower the rural to access local and international markets.

## AUTHOR CONTRIBUTIONS

**Eunice Achiro:** Conceptualization (lead); data curation (lead); formal analysis (lead); funding acquisition (lead); investigation (lead); methodology (lead); project administration (lead); resources (lead); software (lead); supervision (lead); validation (lead); visualization (lead); writing – original draft (lead); writing – review and editing (lead). **Lawrence Okidi:** Conceptualization (supporting); data curation (supporting); formal analysis (supporting); funding acquisition (supporting); investigation (supporting); methodology (supporting); project administration (supporting); resources (supporting); software (supporting); supervision (supporting); validation (supporting); visualization (supporting); writing – original draft (supporting); writing – review and editing (supporting). **Richard Echodu:** Conceptualization (supporting); data curation (supporting); formal analysis (supporting); funding acquisition (supporting); investigation (supporting); methodology (supporting); project administration (supporting); resources (supporting); software (supporting); supervision (supporting); validation (supporting); visualization (supporting); writing – original draft (supporting); writing – review and editing (supporting). **Simon Peter Alarakol:** Conceptualization (supporting); data curation (supporting); formal analysis (supporting); funding acquisition (supporting); investigation (supporting); methodology (supporting); project administration (supporting); resources (supporting); software (supporting); supervision (supporting); validation (supporting); visualization (supporting); writing – original draft (supporting); writing – review and editing (supporting). **Prossy Nassanga:** Conceptualization (supporting); data curation (supporting); formal analysis (supporting); funding acquisition (supporting); investigation (supporting); methodology (supporting); project administration (supporting); resources (supporting); software (supporting); supervision (supporting); validation (supporting); visualization (supporting); writing – original draft (supporting); writing – review and editing (supporting). **Duncan Ongeng:** Conceptualization (equal); data curation (supporting); formal analysis (supporting); funding acquisition (equal); investigation (supporting); methodology (supporting); project administration (supporting); resources (supporting); software (supporting); supervision (supporting); validation (equal); visualization (supporting); writing – original draft (supporting); writing – review and editing (equal).

## CONFLICT OF INTEREST STATEMENT

All authors declare that there is no conflict of interest in this article.

## ETHICS STATEMENT

The study did not involve any animal or human testing. Also, the study protocol and procedures were ethically reviewed and approved by the Gulu University Research Ethical Committee (GUREC 02719) and the Uganda National Council of Science and Technology (SS 4958).

## Data Availability

The data that support the findings of this study are available from the corresponding author upon reasonable request.
